# Young Adult Perspectives on Artificial Intelligence–Based Medication Counseling in China: Discrete Choice Experiment

**DOI:** 10.2196/67744

**Published:** 2025-04-09

**Authors:** Jia Zhang, Jing Wang, JingBo Zhang, XiaoQian Xia, ZiYun Zhou, XiaoMing Zhou, YiBo Wu

**Affiliations:** 1 Shandong Provincial Hospital Affiliated to Shandong First Medical University Jinan China; 2 School of Public Health Peking University Beijing China; 3 Beijing University of Chinese Medicine Evidence Based Medicine Research Center Beijing China; 4 Department of Public Health, Environments & Society London School of Hygiene and Tropical Medicine London United Kingdom; 5 Xiangya School of Nursing Central South University Changsha China; 6 Department of Research Shandong Provincial Hospital Affiliated to Shandong First Medical University Jinan China

**Keywords:** artificial intelligence, medication counseling services, discrete choice experiment, willingness to pay

## Abstract

**Background:**

As artificial intelligence (AI) permeates the current society, the young generation is becoming increasingly accustomed to using digital solutions. AI-based medication counseling services may help people take medications more accurately and reduce adverse events. However, it is not known which AI-based medication counseling service will be preferred by young people.

**Objective:**

This study aims to assess young people’s preferences for AI-based medication counseling services.

**Methods:**

A discrete choice experiment (DCE) approach was the main analysis method applied in this study, involving 6 attributes: granularity, linguistic comprehensibility, symptom-specific results, access platforms, content model, and costs. The participants in this study were screened and recruited through web-based registration and investigator visits, and the questionnaire was filled out online, with the questionnaire platform provided by Questionnaire Star. The sample population in this study consisted of young adults aged 18-44 years. A mixed logit model was used to estimate attribute preference coefficients and to estimate the willingness to pay (WTP) and relative importance (RI) scores. Subgroups were also analyzed to check for heterogeneity in preferences.

**Results:**

In this analysis, 340 participants were included, generating 8160 DCE observations. Participants exhibited a strong preference for receiving 100% symptom-specific results (β=3.18, 95% CI 2.54-3.81; *P*<.001), and the RI of the attributes (RI=36.99%) was consistent with this. Next, they showed preference for the content model of the video (β=0.86, 95% CI 0.51-1.22; *P*<.001), easy-to-understand language (β=0.81, 95% CI 0.46-1.16; *P*<.001), and when considering the granularity, refined content was preferred over general information (β=0.51, 95% CI 0.21-0.8; *P*<.001). Finally, participants exhibited a notable preference for accessing information through WeChat applets rather than websites (β=0.66, 95% CI 0.27-1.05; *P*<.001). The WTP for AI-based medication counseling services ranked from the highest to the lowest for symptom-specific results, easy-to-understand language, video content, WeChat applet platform, and refined medication counseling. Among these, the WTP for 100% symptom-specific results was the highest (¥24.01, 95% CI 20.16-28.77; US $1=¥7.09). High-income participants exhibited significantly higher WTP for highly accurate results (¥45.32) compared to low-income participants (¥20.65). Similarly, participants with higher education levels showed greater preferences for easy-to-understand language (¥5.93) and video content (¥12.53).

**Conclusions:**

We conducted an in-depth investigation of the preference of young people for AI-based medication counseling services. Service providers should pay attention to symptom-specific results, support more convenient access platforms, and optimize the language description, content models that add multiple digital media interactions, and more refined medication counseling to develop AI-based medication counseling services.

## Introduction

Artificial intelligence (AI) is revolutionizing health care by improving the quality of care and facilitating personalized patient engagement. Tools such as virtual assistant chatbots and wearable devices provide real-time support and continuous health monitoring, leading to improved patient outcomes [1-5]. According to the Microsoft-International Data Corporation study in March 2024 [6], 79% of the health care organizations were using AI technology. Research demonstrates that integrating AI with clinicians significantly boosts diagnostic efficiency and accuracy [7], and AI-driven voice or video assistance has proven effective in patient education [8]. In addition, AI helps simplify medical materials, making them easier to understand [9,10], and holds promise for detecting emotional cues in patient interactions, which can enhance communication [11]. These advancements not only streamline health care processes and reduce errors but also contribute to better patient care and have the potential to drive public health improvements and economic growth.

The combination of AI in health consulting has proven to be highly effective. AI can formulate personalized consultations by addressing users’ problems more precisely through intelligent triage and multi-round interactions. Additionally, AI aids doctors in diagnosis and treatment, while assisting patients in managing their health via deep learning and big data analytics [12,13]. AI health consulting services also offer 24/7 availability, supporting various interaction methods such as voice, video, and text, allowing users to benefit from an efficient, intuitive, and enriched service experience. Notable successes include the AI-based breast screening system developed by Google Health and KDDI X-Doctor system [12]. However, limitations have been identified, including concerns about the lack of empathy and interpersonal connection in AI consultations [14]. The Bloomberg Daily Labor Report reports on US health care providers’ use of AI tools to ease administrative burdens and staffing shortages and on risks violating health privacy laws. In addition, studies have shown that AI generates false reports for conceivable diseases, raising concerns about AI dependency and the accuracy of AI-generated results [15,16]. These considerations underscore the importance of understanding user perceptions in AI-assisted health care interventions.

AI-based medication counseling is based on the success of telepharmacy [17,18]. It is a specialized application that uses advanced algorithms to provide personalized guidance for medication management [19,20]. This approach develops an optimized treatment plan by analyzing the patient’s medical history and risk factors. The AI system provides tailored advice on medication use, potential side effects, and drug interactions, ultimately improving patient medication safety and adherence. Web-based counseling has also gained widespread adoption during the COVID-19 period. This suggests that the combination of AI and medication counseling has considerable potential and reliability [21-24].

There is still a gap in the application of AI-based medication counseling services in China, and there is a lack of research on how user preferences affect the effectiveness and acceptance of these systems. In China, the 5 main applications of AI in the health care industry are in medical imaging, assisted diagnosis, drug development, health management, and disease prediction [13]. Although AI technology has made great progress in the medical field [25], the algorithms and models still need to be further improved. AI-based medication counseling services are in their infancy in China, with low market acceptance, and some patients and doctors have insufficient trust in AI, which prevents the service from being widely promoted in the short term [26]. Studies outside China on the preferences of the youth population for health counseling in health care [27,28] have shown that information technology has great potential in helping youth with the management of their personal health. Medication adherence in young individuals cannot be improved by education alone. Increasing the number of behavioral components (eg, monitoring and goal setting, rewards, problem solving, linking medication taking to established procedures) may produce better results. Therefore, we started with established procedures to find out the preferred usage patterns of young people and to fill any gap. Solving these problems is crucial for promoting the combination and popularization of AI and medication counseling in China, and to solve these problems, we aimed to perform a survey among young people. In terms of the generalizability of integrating AI with medication counseling services, young people are the mainstay of adopting new digital health care tools and technologies, and their enthusiasm for technology makes them early adopters of digital health care solutions [29,30]. Most young people are highly receptive to AI and have experience using AI to assist in problem solving. In addition, this age group faces unique challenges, including employment pressures, continuing education, and limited free time. Counseling services that are available anytime, anywhere, and 24/7 for the young population not only saves time, but also has the potential to increase the frequency of medication-related health counseling, improve medication knowledge among the young population [31,32], and avoid the potential harm caused by adverse reactions and unregulated use of medications [33-36].

Additionally, patient preferences are critical, as the success of an AI-based medication counseling service depends on how well it matches the patient’s preferred approach, communication style, and information delivery method. Young people’s willingness to use digital health services can be affected by certain factors: first, privacy breaches and data insecurity, which are the main concerns of users, and second, poor services provided by digital health care, which may also reduce their willingness to use [37-39]. Given young people’s reliance on digital tools, it is likely that their acceptance of AI solutions will be significantly improved if the solutions match their preferences and needs. Understanding their specific preferences and behaviors is therefore critical to tailoring effective and engaging health interventions for this tech-savvy group. Our study aims to explore the preferences of young Chinese people for an AI-based medication counseling service through a discrete choice experiment (DCE) to accelerate the use and popularization of this service in China and to improve patient safety and adherence to medications.

## Methods

### Ethics Approval

The study was approved by the ethics committee of Dongying People’s Hospital (DYYX-2023-168). Information about this study was explained to the participants before the start of the study, and consent was obtained and signed online by each participant for informed consent. Participants completed a web-based questionnaire. Data were anonymized. Participants who completed the experiment were compensated with gifts of approximately ¥5 (US $1 = ¥7.09).

### DCEs

In this study, we used a DCE to quantify the preferences of a youth population for attributes of an AI-based medication counseling service. DCEs are a widely used technique in health economics [40-43]. DCEs are attribute-based benefit measures that present individuals with hypothetical scenarios. It is assumed that respondents use an AI-based medication counseling service for guidance on their medication behaviors and questions. The service used in a given scenario was characterized by specific attributes; the respondent was repeatedly asked to choose among a multiple-option set based on 6 attributes, and the respondent’s assessment of the service depended on the level of these 6 attributes. In examining patient preferences and the willingness to pay (WTP), DCEs were well placed to capture preferences from the patient’s perspective in order to derive recommendations on how to optimize health services from the patient’s perspective, which can be an important factor in ensuring that optimal health services are provided in a given situation [44,45].

### Selection of Attributes and Levels

The determination of attributes and levels in DCEs is a crucial step and was performed as follows: first, conducting literature reviews or expert interviews to develop the initial attributes; second, streamlining data to avoid respondent fatigue (with a recommended upper limit of 6-7 attributes; excessive attributes may increase the burden on the respondent, thus affecting the quality of the respondent’s answer); and third, eliminating inappropriate attributes through further literature review and refining their wording [46-48].

In the initial phase of our study, we tackled key issues in pharmacy services by reviewing literature on AI integration in health care and pharmacy, followed by group discussions and in-depth interviews with clinical pharmacists. Subsequently, to deepen our understanding, it was hypothesized that patients’ preference or satisfaction with AI-based medication counseling services would be influenced by specific attributes identified in this experiment, and based on this hypothesis, 15 respondents without a medical-related background were selected to explore their primary needs in AI-based medication counseling services. Finally, after consulting with relevant experts, respondents ranked their preferences for the identified attributes.

Based on this comprehensive process, 6 key attributes were identified for our experiment: (1) granularity, (2) linguistic comprehensibility, (3) symptom-specific results, (4) access platforms, (5) content model, and (6) costs. The levels for each attribute were assumed and set in a specific order; for instance, the symptomatic nature of the results ranged from 60% to 100%. Table 1 lists each attribute and its corresponding levels. The above 6 attributes were combined with AI, which can mimic human decision-making to communicate with patients and provide general or refined medication counseling services. The patient’s condition can be evaluated by processing large amounts of complex data, enabling the creation of personalized medication plans, including audio or video outputs based on patient needs. Detailed explanations of the attributes and levels are shown in Multimedia Appendix 1.

**Table 1:** Discrete choice experiment attributes and attribute levels for artificial intelligence–based medication counseling service.

Attributes	Levels
Granularity	General Refined
Linguistic comprehensibility	Easy General Difficult
Symptom-specific results	60% 70% 80% 90% 100%
Access platforms	Websites App WeChat applet Hospital official accounts
Content model	Text Phonetics Video
Costs (¥)^a^	0 5 10 15 20

^a^US $1 = ¥7.09.

### Questionnaire and DCE Design

In the design of DCEs, 2 basic principles were observed: balance and orthogonality [49]. Balance indicates that each attribute level occurs with equal frequency in the attributes, for example, level 1 in attribute 1 equals to level 2 in attribute 2. Orthogonality indicates that each pair of levels occurs with equal frequency in all attribute pairs. We used STATA (version 16; StataCorp LLC) for the orthogonality design, which produced 24 choice sets that were randomly divided into 3 versions, each containing 8 choice sets. To prevent participants from having no preferred option when choosing between products A and B, the option “neither” was set. Each choice set had a total of 3 options: offer A, offer B, and neither. Test-retest choice sets were added (ie, respondents were asked to consider the same choice set twice) to check for internal consistency. As a result, each version contained a total of 9 choice sets.

**Figure 1:**
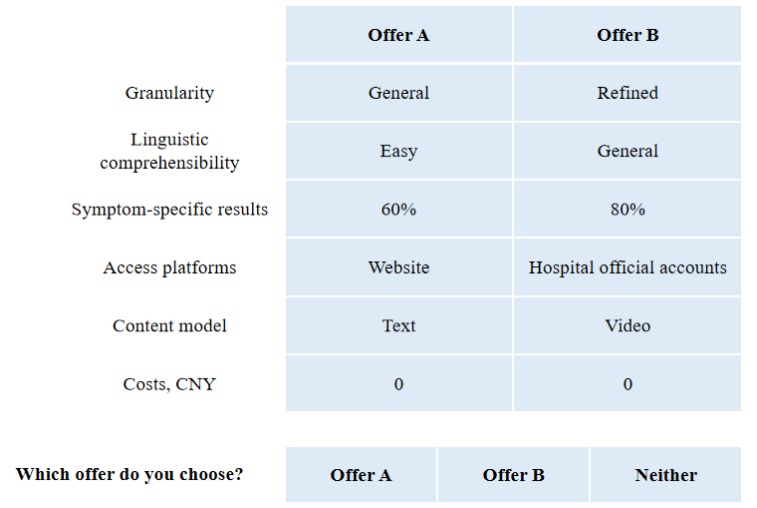
An example of discrete choice experiment selection sets. US $1 = ¥7.09.

The questionnaire consisted of 2 parts. In the first part, known as the demographic question, we aimed to assign basic information about the respondents: age, gender, highest level of education, income, general place, and status of residence. In the second part, there were 9 tasks (including a consistency check option); an example of the tasks is presented in Figure 1.

### Sample Size Calculation

The formula for determining the minimum sample size for a DCE [47] was based on the thumb rule of Johnson and Orme [50]: N≥500 c/ta, where c is the maximum number of levels containing the attribute, t is the number of option sets, and a is the number of options in each option set (including the exit option) [51]. In this experiment, c=5, *t*=24, and a=3. Calculating the sample size according to the above formula gives a minimum sample size of 312 for this experiment.

### Sample Collection and Quality Control

For this study, in terms of sampling methodology, we proceeded in 3 stages, and questionnaires were collected using the Questionnaire Star platform. In the first stage, we identified 3 cities in Shandong province based on their gross domestic product level and city rank: Jinan (2nd), Dongying (9th), and Tai’an (12th). This allows our findings to cover regions with different levels of economic development as a way to ensure the comprehensiveness and fairness of data collection. In the second stage, we considered young people as the survey population in this study. In a survey conducted in 2023 among Chinese college students (with 7055 valid questionnaires), more than 84.88% of the college students reported that they had used AI tools and that they were interested in emerging technology products; therefore, we controlled the survey location by selecting only 2 neighborhoods and a university to perform the survey. The third stage was to perform convenience sampling. Questionnaire collection was performed through 2 methods by the investigators: (1) professional investigators conducted site visits to designated locations to administer the questionnaires, ensuring strict adherence to the inclusion and exclusion criteria to maintain the quality of the data collected, and (2) investigators also posted recruitment information on a web-based platform. Participants who met the location and eligibility criteria were subsequently contacted online by the investigators, who provided detailed explanations regarding the questionnaire and its content. Participants in this study were required to meet the following conditions and sign informed consent: (1) aged between 18 and 44 years [52], (2) can complete the web-based questionnaire on their own or with the help of an investigator, and (3) able to understand the meaning of each entry in the questionnaire. The data collection period was from October to December 2023, and finally, 389 questionnaires were distributed.

To ensure the quality of the responses, we provided centralized training to the enumerators prior to the official launch of the survey, including a detailed explanation of DCE and how to communicate with the respondents. Only those who passed the training were allowed to join the survey team. When the survey was conducted, the investigator explained the questions to the respondents one by one, and some respondents encountered difficulties in completing the questionnaire on their own, for example, operational inconvenience (login restriction, poor network causing slow loading, too many questions making the respondents fatigued, outdated functionality in respondents’ cell phones to load questionnaires and fill them out online). In such cases, the investigator verbally explained the questions to the respondents emphasizing the important parts in order to assist in completing the questionnaire and to ensure the authenticity and validity of the questionnaire. Each respondent was informed that the questionnaire was completely confidential before taking the survey. Questionnaires that met the following exclusion criteria were discarded: (1) those completed in less than 400 seconds, (2) those that failed the consistency check, and (3) those in which all options in the DCE section were identical. After applying these exclusion criteria, a total of 340 valid questionnaires remained, resulting in a response rate of 87.4% (340/389).

### Data Analysis

STATA version 16 was used for the statistical analysis, and we used a random parameter logit model to assess people’s preferences in choosing an AI-based medication counseling service. During the model estimation process, we utilized 500 Halton draws for simulation to enhance the accuracy and efficiency of the integration. Furthermore, in the random parameter logit model, we assumed that the parameters have a latent normal distribution to capture the heterogeneity in preferences among individuals. The advantage of the random parameter logit model is that it allows the parameters to vary randomly across individuals, portraying individual heterogeneity through the distribution of the parameters and better capturing the variability of preferences between individuals. We assumed that all coefficients of the attribute level were random distribution and freely correlated. Cost was treated as a continuous variable in the model to facilitate the calculation of WTP, that is, the relative monetary value that the youth population assigns to various aspects of AI-based medication counseling service. The ratio of regression coefficients of different attributes indicates marginal substitution rates. The ratio of other attributes’ coefficients to the cost coefficient reveals the population’s WTP, applicable across various contexts to assess preferences for different attributes and levels [53-55]. A monetary evaluation of the attributes was conducted as follows. The WTP for a single level was calculated by dividing the estimated β of the attribute level by the negative of the estimated costs β. A positive estimate means that people were willing to pay for that level, while a negative estimate indicates unwillingness to pay. Although cost was treated as a continuous variable for WTP calculations, it was considered a categorical variable for the calculation of relative importance (RI). In order to determine each attribute’s RI in relation to all other characteristics or levels, the marginal utility range of each attribute was divided by the total of all attribute ranges. Moreover, we performed subgroup analyses by income and education levels and calculated WTP values by using a mixed logit model to evaluate the influence of demographic factors on cost sensitivity.

## Results

### Participants’ Characteristics

Among the 340 participants included in this study, 161 (47.4%) were males and 179 (52.6%) were females; 262 (77%) participants were in the age range of 18-24 years. Approximately 80.6% (274/340) of the participants were not living alone and 79.4% (270/340) were unmarried. Approximately 85.9% (292/340) of the participants had tertiary education or above. Specific data are shown in Table 2.

**Table 2:** Sociological characteristics of the participants (N=340).

Characteristics		Values, n (%)
**Age (years)**		
	18-24	262 (77)
	25-30	36 (10.6)
	31-44	42 (12.4)
**Sex**		
	Male	161 (47.4)
	Female	179 (52.6)
**Educational attainment**		
	High school and below	48 (14.1)
	College and above	292 (85.9)
**Average monthly household income (¥)^a^**		
	≤2000	44 (12.9)
	2000-4000	97 (28.5)
	4000-6000	100 (29.4)
	≥6000	99 (29.1)
**Residence**		
	City	293 (86.2)
	Countryside	47 (13.8)
**Household registration**		
	Nonagricultural	122 (35.9)
	Agricultural	218 (64.1)
**Matrimonial status**		
	Married	70 (20.6)
	Unmarried/divorced/widowed	270 (79.4)
**Living status**		
	Living alone	66 (19.4)
	Not living alone	274 (80.6)

^a^US $1 = ¥7.09.

### Results From the Mixed Logit Model

**Figure 2:**
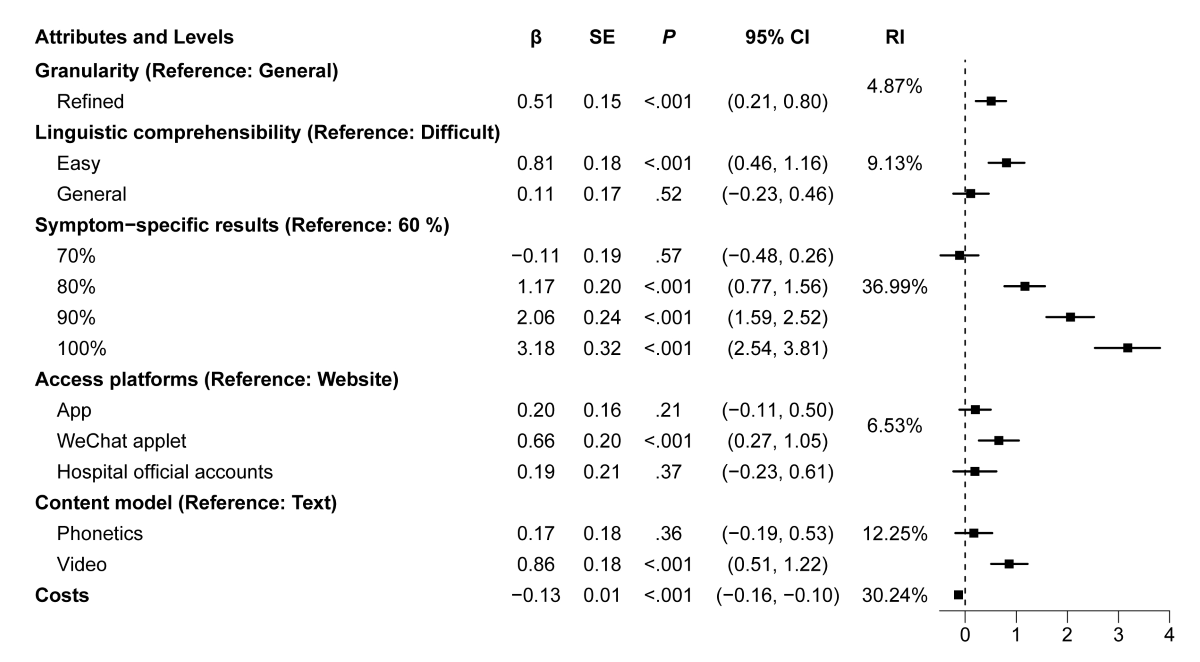
Mixed logit model regression of preferences of artificial intelligence–based medication counseling service. Model fit: log likelihood=–1928.0508; R2=0.1261; Akaike Information Criterion/N=1.249; Bayesian Information Criterion/N=1.280. RI: relative importance.

According to the mixed logit model results shown in Figure 2, all attributes were statistically significant, and there were varied preferences across attribute levels. We found that users were most concerned about whether the service was free or not. Preferred service attributes included refined medication counseling services (with general medication counseling services as the reference; β=0.51; *P*<.001), easy-to-understand language (with difficulty in understanding language as the reference; β=0.81; *P*<.001), 100% symptom-specific results (with 60% symptom-specific results of the results as the reference; β=3.18; *P*<.001), 80% symptom-specific results (β=1.17; *P*<.001), 90% symptom-specific results (β=2.06; *P*<.001), through WeChat applets (website access as reference; β=0.66; *P*<.001), and content model in video form (text as reference; β=0.86; *P*<.001).

The 100% level of symptom-specific results was strongly preferred by the respondents, and the same answer was obtained during the face-to-face conversations with the respondents. These coefficients reflect the RI of each attribute level to user preferences; *P* values test the statistical significance of the coefficients; and standard errors and 95% CIs provide credibility to the coefficient estimates. An in-depth analysis of these preferences can provide guidance for the design and optimization of AI services.

Among the 6 attributes in this study, the most important factor influencing the young population to make a choice was the symptom-specific results (RI=36.99%), followed by cost (RI=30.24%), access platforms (RI=6.53%), content models (RI=12.25%), linguistic comprehensibility (RI=9.13%), and granularity (RI=4.87%).

### WTP

As shown in Table 3, participants were willing to pay an additional ¥3.84 for fine-tuned medication counseling. For linguistic comprehensibility, participants were willing to pay an additional ¥6.14 for clearer language delivery. For symptom-specific results, participants were willing to pay ¥24.01 more for 100% results, ¥15.55 more for 90% results, and ¥8.82 more for 80% results. For access platforms, respondents were willing to pay an additional ¥4.99 to access the counseling service via WeChat applet. For the content model, participants were willing to pay ¥6.54 more for video-based content delivery. We evaluated the influence of demographic factors on cost sensitivity by using subgroup analyses, with WTP estimated based on the results. Specific results are shown in Multimedia Appendix 2.

**Table 3:** Willingness to pay analysis of participants’ perceptions of artificial intelligence–based medication counseling service.

Attributes and levels			Willingness to pay, β (95% CI)
**Granularity**			
	General	Reference	
	Refined	3.84 (1.74 to 6.00)	
**Linguistic comprehensibility**			
	Difficult	Reference	
	Easy	6.14 (3.50 to 9.32)	
	General	0.85 (–1.67 to 3.72)	
**Symptom-specific results**			
	60%	Reference	
	70%	–0.83 (–3.66 to 2.14)	
	80%	8.82 (6.16 to 11.82)	
	90%	15.55 (12.41 to 19.45)	
	100%	24.01 (20.16 to 28.77)	
**Access platforms**			
	Website	Reference	
	App	1.50 (–0.81 to 3.86)	
	WeChat applet	4.99 (2.22 to 7.90)	
	Hospital official accounts	1.45 (–1.75 to 4.70)	
**Content model**			
	Text	Reference	
	Phonetics	1.28 (–1.43 to 4.16)	
	Video	6.54 (4.18 to 9.06)	

## Discussion

### Principal Findings

In this study, we analyzed Chinese youths’ preferences for AI-based medication counseling services, and we identified 3 standout attributes: symptom-specific results, content model, and linguistic comprehensibility [56]. Respondents showed a strong preference for highly symptom-specific results, which were correlated with higher WTP. Based on our survey data, we suggest that AI-based medication counseling services should prioritize the enhancement of symptom-specific outcomes, which may help improve user satisfaction and WTP.

A study [57] has shown that AI technology can improve diagnostic accuracy in certain areas, but the effects may vary in different contexts. Regarding content models, digital formats such as voice and video are preferred over text probably because they are more engaging and easier to comprehend [58]. Text explanations can make patients feel passive because of varying levels of understanding. Video explanations enriched with health science information could improve patient attention and health literacy [58-60].

For linguistic comprehensibility, plain language is preferred, with clear and understandable explanations being critical. AI’s lack of emotional response in counseling was noted as a concern. AI emotion recognition has the potential to improve communication in the future, but more research and data are needed at this stage to validate this hypothesis—maybe the interaction could be made more empathetic and reassuring.

Respondents demonstrated a strong preference for WeChat applets over websites as access platforms because of their simplicity and user-friendliness. Further research could explore the factors underlying this preference. WeChat applets offer smooth, user-friendly access, while web pages may contain disinformation and are less convenient to save and revisit. WeChat applets are increasingly popular due to their simplicity, lack of installation requirement, large user base, and low development costs [61]. Preference for WeChat applets and easy-to-understand language may reflect the preferences of the respondents for simple everyday use across a number of previously indicated attribute levels.

In terms of medication guidance, respondents expect AI to provide more detailed and refined advice compared to traditional methods. However, the one-question, one-answer format of AI services can limit the patients in asking follow-up questions or explore further, thus causing them to overlook important issues. Comprehensive counseling addressing all aspects of drug use is essential.

Finally, cost was also a key factor considered by the respondents, with a significant preference for low-cost or free medication counseling. This is consistent with the results of previous related DCE studies for pharmacy services [62,63]. Respondents were willing to pay ¥24.01 for 100% symptom-specific results. This implied that the respondents were not concerned about the high cost of counseling services for highly symptomatic patients. It is possible that the respondents anticipate receiving more precise counseling outcomes and, thus, a better quality of health care. According to the subgroup analysis results, high-income individuals demonstrated greater payment capacity and were more willing to pay a premium for higher-quality health care services. Additionally, high-income groups tended to place more value on personalized and precise services. Similarly, individuals with higher education levels often had greater demands for information accessibility and comprehension, reporting easy-to-understand language and video content models more effective in meeting their needs. Higher income and education levels were associated with a greater WTP for premium services and a higher value placed on personalized services.

Symptom-specific results and costs formed the largest portion of RI, and we believe that symptom-specific results are directly related to treatment outcomes and patient satisfaction. Of the attributes we provided, only symptom-specific results and costs were quantified by specific numbers, which allowed respondents to compare and assess the extent of services more intuitively when making choices. This suggests that young people are likely to combine economic pragmatism, that is, finding the optimal solution with limited resources when making decisions.

### Recommendations

A well-designed policy framework is essential for the successful integration of AI-based medication counseling services into health care systems. Policymakers need to consider the key findings regarding symptom-specific results, content model, linguistic comprehensibility, and the need for cost-effective, user-friendly platforms.

#### Enhancing Symptom-Specific Results

Policymakers should encourage the integration of advanced algorithms and data analytics to improve the precision of AI-generated recommendations. It is crucial to tailor these services to address individual health conditions and needs more effectively.

#### Improving Content Model Formats

Incorporating video and voice features into AI-based counseling platforms could enhance user engagement, foster better comprehension of health information, and improve overall health literacy. These formats can be particularly effective in communicating complex medical advice in a user-friendly manner.

#### Ensuring Linguistic Comprehensibility

The preference for clear, plain language indicates the importance of designing AI-based medication counseling services that prioritize linguistic comprehensibility. Policymakers should mandate the use of simple, understandable language in all patient-facing content. Additionally, AI systems should be capable of adjusting their language to suit the linguistic preferences of diverse users. The inclusion of culturally sensitive language as well as the ability to offer services in multiple languages and dialects should be prioritized to accommodate the needs of different populations.

#### Incorporating Emotional Intelligence Into AI Interactions

The lack of emotional response in AI counseling was identified as a gap in the current services. Policymakers should advocate for the integration of emotion recognition capabilities in AI systems. These systems could analyze user language, tone, and context to detect emotions such as anxiety or confusion. Based on this analysis, AI can provide appropriate emotional responses, such as reassuring or encouraging messages, thereby enhancing the patient-centered nature of the service. This addition could improve user satisfaction and foster a more empathetic interaction with technology.

#### Cost Considerations in Service Design

Policymakers should focus on designing AI-based counseling services that are affordable, ensuring that cost does not become a barrier to access. At the same time, it is important to acknowledge that users are willing to pay for higher quality services, especially those that offer precise symptom-specific guidance. Subsidies or tiered pricing models could be introduced to ensure the affordability of these services while maintaining high quality.

#### Data Privacy and Security

Policymakers need to create strict guidelines to protect patient data in AI-based medication counseling services. This includes obtaining informed consent, securely storing and processing data, and ensuring privacy standards are met to build trust and encourage adoption.

### Limitations

This study has several limitations. First, this study was conducted on a specific population: young adults aged 18-44 years. Future product designs should expand the scope of this study, considering the perspectives of various demographic groups to maximize the benefits of AI-based medication counseling service. For example, studying preferences among older populations or testing AI-based services with different health conditions could provide a broader view of AI’s role in medication counseling. Addressing the specific needs of different groups will be the focus of our next efforts. Second, this study uses convenience sampling in the third step of sampling, and only 1 province was selected to conduct the survey; the data can be used in the future to improve the representativeness of the sample in a wider range of areas by increasing the sample size, employing more random sampling techniques, and extending the data collection period.

### Conclusion

This study aims to determine young respondents’ preferences for using AI-based medication counseling services in China. Our findings indicate that the 6 attributes that respondents found most appealing were 100% symptom-specific results, refined medication counseling, WeChat applet platforms, easily understandable content, video content mode, and no payment required. In summary, when young people choose AI-based medication counseling services, policymakers should pay more attention to considering economic practicality and personalized needs when designing services for young people so as to meet their expectations for high-quality and reasonably costly health care services, and better understanding their needs and promote AI-based medication counseling as a way to solve problems, increase engagement and medication adherence, and establish them as the first to benefit from AI-pharmacy integration and improve the safety of medication and medication literacy of users. These findings will provide valuable insights for policymakers to drive the implementation of AI-based medication counseling services.

## Appendix

Multimedia Appendix 1 Explanations of the study attributes and levels.

Multimedia Appendix 2 Questionnaire used in this study.

## Abbreviations

AI: artificial intelligence
DCE: discrete choice experiment
RI: relative importance
WTP: willingness to pay

## Data Availability

The data that support the findings of this study are available on request from the corresponding author upon reasonable request.

## References

[1] Xie Y, Seth I, Hunter-Smith DJ, Rozen WM, Ross R, Lee M (2023). Aesthetic surgery advice and counseling from artificial intelligence: a rhinoplasty consultation with ChatGPT. Aesthetic Plast Surg.

[2] Sandeep Ganesh G, Kolusu AS, Prasad K, Samudrala PK, Nemmani KVS (2022). Advancing health care via artificial intelligence: From concept to clinic. Eur J Pharmacol.

[3] Altamimi I, Altamimi A, Alhumimidi AS, Altamimi A, Temsah M (2023). Snakebite advice and counseling from artificial intelligence: an acute venomous snakebite consultation with ChatGPT. Cureus.

[4] Kricka LJ, Polevikov S, Park JY, Fortina P, Bernardini S, Satchkov D, Kolesov V, Grishkov M (2020). Artificial intelligence-powered search tools and resources in the fight against COVID-19. EJIFCC.

[5] Li Y, Li Y, Wei M, Li G (2024). Innovation and challenges of artificial intelligence technology in personalized healthcare. Sci Rep.

[6] Artificial intelligence (AI) reshaping healthcare industry with unimaginable potential. GlobeNewsWire.

[7] Liu T, Tsang W, Xie Y, Tian K, Huang F, Chen Y, Lau O, Feng G, Du J, Chu B, Shi T, Zhao J, Cai Y, Hu X, Akinwunmi B, Huang J, Zhang CJP, Ming W (2021). Preferences for artificial intelligence clinicians before and during the COVID-19 pandemic: discrete choice experiment and propensity score matching study. J Med Internet Res.

[8] Kinoshita K, Maruyama T, Kobayashi N, Imanishi S, Maruyama M, Ohira G, Endo S, Tochigi T, Kinoshita M, Fukui Y, Kumazu Y, Kita J, Shinohara H, Matsubara H (2024). An artificial intelligence-based nerve recognition model is useful as surgical support technology and as an educational tool in laparoscopic and robot-assisted rectal cancer surgery. Surg Endosc.

[9] Şahin MF, Topkaç Erdem Can, Şeramet S, Özcan Rıdvan, Akgül Murat, Yazıcı CM, Doğan (2024). Still using only ChatGPT? The comparison of five different artificial intelligence chatbots' answers to the most common questions about kidney stones. J Endourol.

[10] Li G, Lin MX, Cui D, Mathews PM, Akpek EK (2025). Enhancing online cataract surgery patient education materials through artificial intelligence. Can J Ophthalmol.

[11] Rathje S, Mirea D, Sucholutsky I, Marjieh R, Robertson CE, Van Bavel JJ (2024). GPT is an effective tool for multilingual psychological text analysis. Proc Natl Acad Sci U S A.

[12] Improving breast cancer screening with artificial intelligence. Google Health.

[13] Jiang Fei, Jiang Yong, Zhi Hui, Dong Yi, Li Hao, Ma Sufeng, Wang Yilong, Dong Qiang, Shen Haipeng, Wang Yongjun (2017). Artificial intelligence in healthcare: past, present and future. Stroke Vasc Neurol.

[14] Haidet P, Melro CM, Fecile MLE, Jarecke JLT, Moniz T, Cooper AB (2024). Shared decision making reimagined. Patient Educ Couns.

[15] Stephan D, Bertsch A, Burwinkel M, Vinayahalingam S, Al-Nawas B, Kämmerer Peer W, Thiem DG (2024). AI in dental radiology-improving the efficiency of reporting with ChatGPT: comparative study. J Med Internet Res.

[16] Yokokawa D, Yanagita Y, Li Y, Yamashita S, Shikino K, Noda K, Tsukamoto T, Uehara T, Ikusaka M (2024). For any disease a human can imagine, ChatGPT can generate a fake report. Diagnosis (Berl).

[17] Win AZ (2017). Telepharmacy: Time to pick up the line. Res Social Adm Pharm.

[18] Le T, Toscani M, Colaizzi J (2020). Telepharmacy: a new paradigm for our profession. J Pharm Pract.

[19] Robinson CL, D'Souza RS, Yazdi C, Diejomaoh EM, Schatman ME, Emerick T, Orhurhu V (2024). Reviewing the potential role of artificial intelligence in delivering personalized and interactive pain medicine education for chronic pain patients. J Pain Res.

[20] Ni Z, Peng ML, Balakrishnan V, Tee V, Azwa I, Saifi R, Nelson LE, Vlahov D, Altice FL (2024). Implementation of chatbot technology in health care: protocol for a bibliometric analysis. JMIR Res Protoc.

[21] Morath B, Chiriac U, Jaszkowski E, Deiß Carolin, Nürnberg Hannah, Hörth Katrin, Hoppe-Tichy T, Green K (2024). Performance and risks of ChatGPT used in drug information: an exploratory real-world analysis. Eur J Hosp Pharm.

[22] Hsu H, Hsu K, Hou S, Wu C, Hsieh Y, Cheng Y (2023). Examining real-world medication consultations and drug-herb interactions: ChatGPT performance evaluation. JMIR Med Educ.

[23] Kunitsu Y (2023). The potential of gpt-4 as a support tool for pharmacists: analytical study using the Japanese National Examination for Pharmacists. JMIR Med Educ.

[24] Abu-Farha R, Fino L, Al-Ashwal FY, Zawiah M, Gharaibeh L, Harahsheh MM, Darwish Elhajji F (2023). Evaluation of community pharmacists' perceptions and willingness to integrate ChatGPT into their pharmacy practice: A study from Jordan. J Am Pharm Assoc (2003).

[25] Wekenborg Magdalena Katharina, Gilbert Stephen, Kather Jakob Nikolas (2025). Examining human-AI interaction in real-world healthcare beyond the laboratory. NPJ Digit Med.

[26] Rong G, Mendez A, Bou Assi E, Zhao B, Sawan M (2020). Artificial intelligence in healthcare: review and prediction case studies. Engineering.

[27] Majeed-Ariss R, Baildam E, Campbell M, Chieng A, Fallon D, Hall A, McDonagh JE, Stones SR, Thomson W, Swallow V (2015). Apps and adolescents: a systematic review of adolescents' use of mobile phone and tablet apps that support personal management of their chronic or long-term physical conditions. J Med Internet Res.

[28] Dean AJ, Walters J, Hall A (2010). A systematic review of interventions to enhance medication adherence in children and adolescents with chronic illness. Arch Dis Child.

[29] Norman CD, Yip AL (2012). eHealth promotion and social innovation with youth: using social and visual media to engage diverse communities. Stud Health Technol Inform.

[30] Knapp AA, Cohen K, Nicholas J, Mohr DC, Carlo AD, Skerl JJ, Lattie EG (2021). Integration of digital tools into community mental health care settings that serve young people: focus group study. JMIR Ment Health.

[31] Newton P, Cabot L, Wilson NHF, Gallagher JE (2011). The graduate entry generation: a qualitative study exploring the factors influencing the career expectations and aspirations of a graduating cohort of graduate entry dental students in one London institution. BMC Oral Health.

[32] Zayts O, Edmonds DM, Kong BCK, Fortune Z (2023). Mental health of new and recent graduates during the university-to-work transition: a scoping review protocol. BMJ Open.

[33] Alshammari TM (2016). Drug safety: The concept, inception and its importance in patients' health. Saudi Pharm J.

[34] Grissinger MC, Kelly K (2005). Reducing the risk of medication errors in women. J Womens Health (Larchmt).

[35] Santell JP, Hicks RW (2005). Medication errors involving geriatric patients. Jt Comm J Qual Patient Saf.

[36] Abdelaziz S, Amigoni A, Kurttila M, Laaksonen R, Silvari V, Franklin BD (2025). Medication safety strategies in European adult, paediatric, and neonatal intensive care units: a cross-sectional survey. Eur J Hosp Pharm.

[37] Zhang L, Hu X (2022). Analysis of the smart medical service model in super-aged society-UR agency as an example. J Healthc Eng.

[38] Almalawi A, Khan AI, Alsolami F, Abushark YB, Alfakeeh AS (2023). Managing Security of Healthcare Data for a Modern Healthcare System. Sensors (Basel).

[39] Zhang H, Liu Y, Gu R (2024). Correlation between psychological traits and the use of smart medical services in young and middle-aged adults: An observational study. World J Psychiatry.

[40] Ryan M, Gerard K (2003). Using discrete choice experiments to value health care programmes: current practice and future research reflections. Appl Health Econ Health Policy.

[41] Cleland J, Porteous T, Skåtun Diane (2018). What can discrete choice experiments do for you?. Med Educ.

[42] Naik-Panvelkar P, Armour C, Saini B (2013). Discrete choice experiments in pharmacy: a review of the literature. Int J Pharm Pract.

[43] McFadden D (1972). Conditional logit analysis of qualitative choice behaviour. Frontiers in Econometrics.

[44] Ostermann J, Mühlbacher Axel, Brown DS, Regier DA, Hobbie A, Weinhold A, Alshareef N, Derrick C, Thielman NM (2020). Heterogeneous patient preferences for modern antiretroviral therapy: results of a discrete choice experiment. Value Health.

[45] Phillips EA, Himmler SF, Schreyögg Jonas (2021). Preferences for e-mental health interventions in Germany: a discrete choice experiment. Value Health.

[46] Coast J, Al-Janabi H, Sutton EJ, Horrocks SA, Vosper AJ, Swancutt DR, Flynn TN (2012). Using qualitative methods for attribute development for discrete choice experiments: issues and recommendations. Health Econ.

[47] Helter TM, Boehler CEH (2016). Developing attributes for discrete choice experiments in health: a systematic literature review and case study of alcohol misuse interventions. J Subst Use.

[48] Ryan M (1999). Using conjoint analysis to take account of patient preferences and go beyond health outcomes: an application to in vitro fertilisation. Soc Sci Med.

[49] Bailey RA (2018). Balance, orthogonality and efficiency factors in factorial design. Journal of the Royal Statistical Society: Series B (Methodological).

[50] Johnson R, Orme B (2003). Getting the most from CBC. Sawtooth Software Research Paper.

[51] de Bekker-Grob EW, Donkers B, Jonker MF, Stolk EA (2015). Sample size requirements for discrete-choice experiments in healthcare: a practical guide. Patient.

[52] Ahmad O, Boschi-Pinto C, Lopez A, Murray C, Lozano R, Inoue M (2000). Age standardization of rates: A new WHO standard. World Health Organization.

[53] Fung LWY, Zhao J, Yan VKC, Blais JE, Chan JCH, Li STH, Shami JJP, Kwan C, Wei Y, Wong CKH, Li X, Chui CSL, Wan EYF, Lai FTT, Tse S, Cowling BJ, Wong ICK, Chan EW (2022). COVID-19 vaccination preferences of university students and staff in Hong Kong. JAMA Netw Open.

[54] Liu S, Liu J, Si L, Ke X, Liu L, Ren Y, Bao S, Li F, Yu Y, Pan Q, Wei Y, Chen Y (2023). Patient preferences for anti-hyperglycaemic medication for type 2 diabetes mellitus in China: findings from a national survey. BMJ Glob Health.

[55] Dhanda DS, Veenstra DL, Regier DA, Basu A, Carlson JJ (2020). Payer preferences and willingness to pay for genomic precision medicine: a discrete choice experiment. J Manag Care Spec Pharm.

[56] Stonerock GL, Blumenthal JA (2017). Role of counseling to promote adherence in healthy lifestyle medicine: strategies to improve exercise adherence and enhance physical activity. Prog Cardiovasc Dis.

[57] Esteva Andre, Kuprel Brett, Novoa Roberto A, Ko Justin, Swetter Susan M, Blau Helen M, Thrun Sebastian (2017). Dermatologist-level classification of skin cancer with deep neural networks. Nature.

[58] Conard S (2019). Best practices in digital health literacy. Int J Cardiol.

[59] Lenczowski E, Tung-Hahn E, Higareda J, McCormick C, Markoff T, Arffa M, Poon E, Lee K, Alam M (2018). Video education to improve recognition of common benign and malignant cutaneous lesions and skin cancer prevention in the public. Int J Womens Dermatol.

[60] Goodman RS, Patrinely JR, Osterman T, Wheless L, Johnson DB (2023). On the cusp: Considering the impact of artificial intelligence language models in healthcare. Med.

[61] Shiferaw KB, Tilahun BC, Endehabtu BF, Gullslett MK, Mengiste SA (2020). E-health literacy and associated factors among chronic patients in a low-income country: a cross-sectional survey. BMC Med Inform Decis Mak.

[62] Sun Q, Wang Y, Wang P, Huang Y, Xi X (2024). Residents preferences for pharmacist-managed clinic in China: a discrete choice experiment. Patient Prefer Adherence.

[63] Porteous T, Ryan M, Bond C, Watson M, Watson V (2016). . Managing minor ailments; the public's preferences for attributes of community pharmacies: A discrete choice experiment. PLoS One.

