# Supplementary material for: Young Adult Perspectives on Artificial Intelligence–Based Medication Counseling in China: Discrete Choice Experiment
Source: J Med Internet Res. 2025 Apr 9;27:e67744. doi: 10.2196/67744 (PMC12018864; doi:10.2196/67744)
Supplement: Multimedia Appendix 1 [file jmir_v27i1e67744_app1.docx]

# Explanations of the study attributes and levels

●**Granularity:**

AI-based medication counseling services are categorized into two approaches based on the level of granularity of the medication guidance provided: general medication counseling and refined medication counseling.

**General:** Through artificial intelligence technology, it provides patients with basic services such as medication advice for common diseases and drug information query. Based on a vast medication database and disease knowledge base, it is possible to swiftly answer patients' generic inquiries regarding drugs, such as drug name, usage, dose, adverse reactions, etc.

**Refined:** It gives patients more precise and tailored drug recommendations when combined with their individual circumstances (genotype, disease status, allergy history, etc.). It analyzes the patient's condition and medication in-depth using artificial intelligence algorithms and machine learning technologies to create a medicine plan that is more suited to the patient's needs. In order to optimize the therapeutic effect, this method also permits timely medication regimen adjustments and real-time monitoring of the patient's pharmaceutical effects.

**●Linguistic comprehensibility:**

Linguistic comprehensibility refers to the extent to which the linguistic information output by an AI system when providing medication counseling services can be accurately and clearly understood by the user. This involves a number of aspects such as the vocabulary, sentence structure, and expressions used by the system, and is intended to ensure that users can access and understand medication counseling information without any barriers.

There are three levels: easy, general, and difficult.

**Easy:** The language used by the AI system is very concise and clear, and the vocabulary and sentence structure are in line with the user's daily language habits. The system is able to clearly explain key information such as medication methods, dosages, precautions, etc., which can be easily understood by the user without extra effort. The system may use colloquial expressions and avoid overly specialized or complex terms. At the same time, the system will also guide the user to understand the information through a clear logical structure and explicit instructions;

**General:** The linguistic information provided by the AI system is relatively clear, but may contain some specialized terms or more complex sentence structures. Users will need to spend some time and effort to understand the information, but this can usually be accomplished within acceptable limits. The system may use some specialized terminology to describe medicines or diseases, but will help the user to understand the meaning of these terms through context or additional explanations. In addition, the system will improve the readability of the information through appropriate segmentation and typography;

**Difficult:** The linguistic information provided by the AI system is complex or obscure, making it difficult for the user to understand or accurately grasp the key information. This may lead to users misinterpreting medication instructions or overlooking important precautions, and the system may use a lot of jargon, complex sentence structures, or expressions that are difficult to understand. In addition, the system may lack a clear logical structure and explicit instructions, making it difficult for users to grasp the overall framework and key points of the information.

●**Symptom-specific results:**

Appropriateness refers to the ability of a medication counseling service to accurately identify a patient's specific symptoms or disease and recommend the appropriate medication or treatment plan.

The degree of matching of medication instructions to the patient's symptoms was categorized into five levels, 60%, 70%, 80%, 90%, and 100%. The level of match increases progressively from 60% to 100%, with 60% being low symptomaticity, where the service has difficulty in accurately identifying the patient's symptoms or recommends medicines or treatments that do not match the patient's symptoms, and 100% being high symptomaticity, where the service is able to accurately identify the patient's symptoms and recommend medicines or treatments that are highly relevant to the patient's symptoms.

**●Access platforms**

The access platform is the channel or interface used by the user to access and use the AI-based medication counseling service. There are four levels: website, APP, WeChat app, and hospital public number.

**Website:** A web platform accessed through an internet browser where users can browse, search, and access medication counseling services, and websites usually have broad compatibility and can be used on a variety of devices and operating systems. At the same time, websites can provide a wealth of information and features, such as detailed drug descriptions, medication guides, and expert advice. However, access to the website may be limited by the network environment and device performance.

**APP:** Refers to applications installed on smartphones or tablets, through which users can directly access and use medication counseling services.APP usually has better user experience and interactivity, and can provide more personalized services and push notifications. In addition, APPs can utilize the hardware features of the device (e.g., camera, microphone, etc.) to enhance the convenience and accuracy of the service. However, the download and installation of APP may take some time and traffic.

**WeChat applets:** Applications that can be used without downloading and installing, allowing users to open and use medication counseling services directly in WeChat. WeChat applets are lightweight, easy to use and ready-to-use, so users don't need to worry about downloading and installation. At the same time, WeChat applets can fully utilize the social function of WeChat, facilitating users to share and discuss medication issues. However, the functions and performance of WeChat small programs may be limited by the WeChat platform.

**Hospital official accounts:** An official account opened by a hospital on WeChat or other social media platforms, which users can follow to obtain medication counseling services. Hospital public numbers are usually closely linked to the hospital's medical resources and service system, and can provide authoritative and professional medication guidance and consultation services. In addition, the public number can also publish hospital news and health information to enhance users' health awareness and trust. However, access to hospital public numbers may be limited by users' social media usage habits

**●Content model:**

The form in which the service provides information.

**Text:** This is the most basic content mode, providing medication advice through textual descriptions

**Phonetics:** Through speech synthesis technology, medication counseling information is conveyed to the user in the form of speech.

**Video:** Using video technology to provide medication counseling services through the combination of vision and hearing, which can include animation, live demonstration and other content to visually display the medication process.

**●Costs:**

This is the out-of-pocket amount that patients will have to pay each time they use what should be an AI-based medication counseling service.

# Supplementary materials 2. Parental, sub-group analyses

Table S1-incom

Table S2-education

Table S1 (A) Participants who with a monthly income of less than 5,000 RMB

|  | Level | β (SE) | 95% CI | | *P* | WTP | 95% CI | |
| --- | --- | --- | --- | --- | --- | --- | --- | --- |
| Granularity | General | Reference | | | | | | |
|  | Refined | 1.04(0.39) | 0.27 | 1.8 | 0.008 | 5.43 | 2.49 | 7.61 |
| Linguistic comprehensibility | Difficult | Reference | | | | | | |
|  | Easy | 0.92(0.35) | 0.24 | 1.6 | 0.008 | 4.82 | 2.56 | 9.26 |
|  | General | -0.1(0.3) | -0.69 | 0.49 | 0.740 | -0.52 | -2.37 | 3.96 |
| Symptom-specific results | 60 % | Reference | | | | | | |
|  | 70 % | -0.41(0.34) | -1.09 | 0.26 | 0.23 | -2.15 | -7.29 | 1.39 |
|  | 80 % | 1.27(0.46) | 0.36 | 2.18 | 0.006 | 6.61 | 4.53 | 11.12 |
|  | 90 % | 2.54(0.89) | 0.80 | 4.27 | 0.004 | 13.24 | 10.25 | 18.06 |
|  | 100% | 3.96(0.91) | 2.17 | 5.74 | <0.001 | 20.65 | 16.09 | 26.64 |
| Access platforms | Websites | Reference | | | | | | |
|  | App | 0.50(0.31) | -0.11 | 1.11 | 0.10 | 2.62 | -1.61 | 5.59 |
|  | WeChat applet | 1,18(0.55) | 0.10 | 2.27 | 0.03 | 6.17 | 1.85 | 8.56 |
|  | Hospital official accounts | 0.81(0.53) | -0.22 | 1.84 | 0.12 | 4.25 | 0.17 | 7.10 |
| Content model | Text | Reference | | | | | | |
|  | Phonetics | 0.19(0.32) | -0.43 | 0.81 | 0.54 | 1.00 | -3.53 | 4.16 |
|  | Video | 1.17(0.37) | 0.43 | 1.90 | 0.002 | 6.08 | 3.25 | 9.89 |
| Costs |  | -.19(0.06) | -0.30 | -0.08 | <0.001 |  | 1.85 | 8.56 |

Table S1(B) Participants who with a monthly income of more than 5,000 RMB

|  | Level | Preference mean difference (SE) | 95% CI | | *P* | WTP | 95% CI | |
| --- | --- | --- | --- | --- | --- | --- | --- | --- |
| Granularity | General | Reference | | | | | | |
|  | Refined | 5.42(2.96) | -0.37 | 11.22 | .07 | 1.84 | -2.92 | 5.39 |
| Linguistic comprehensibility | Difficult | Reference | | | | | | |
|  | Easy | 15(5.95) | 3.34 | 26.66 | .01 | 5.1 | 3.20 | 15.85 |
|  | General | 1.8(2.89) | -3.86 | 7.46 | .53 | 0.61 | -2.08 | 8.47 |
| Symptom-specific results | 60 % | Reference | | | | | | |
|  | 70 % | 8.9(4.10) | 0.85 | 16.94 | .03 | 3.02 | -4.57 | 8.61 |
|  | 80 % | 33.2(12.90) | 7.91 | 58.49 | .01 | 11.29 | 8.39 | 20.13 |
|  | 90 % | 49.9(19.48) | 11.72 | 88.09 | .01 | 16.97 | 15.55 | 30.25 |
|  | 100% | 133.25(51.66) | 32.01 | 234.50 | .01 | 45.32 | 23.01 | 44.03 |
| Access platforms | Websites | Reference | | | | | | |
|  | App | 10.56(4.97) | 0.81 | 20.30 | .03 | 3.59 | -9.64 | 5.51 |
|  | WeChat applet | 10.13(4.87) | 0.59 | 19.66 | .04 | 3.44 | -3.47 | 9.35 |
|  | Hospital official accounts | -1.63(3.26) | -8.02 | 4.74 | .61 | -0.56 | -11.97 | 4.08 |
| Content model | Text | Reference | | | | | | |
|  | Phonetics | 2.44(2.63) | -2.72 | 7.60 | .35 | 0.83 | -7.61 | 6.69 |
|  | Video | 14.18(5.87) | 2.67 | 25.68 | .02 | 4.82 | -2.13 | 10.23 |
| Costs |  | -2.94(1.14) | -5.17 | -.71 | .01 |  |  |  |

Table S2 (A) Participants with higher educational qualifications than post-secondary students

|  | Level | Preference mean difference (SE) | 95% CI | | *P* | WTP | 95% CI | |
| --- | --- | --- | --- | --- | --- | --- | --- | --- |
| Granularity | General | Reference | | | | | | |
|  | Refined | 0.74(0.20) | 0.34 | 1.14 | <0.001 | 6.21 | 3.24 | 9.33 |
| Linguistic comprehensibility | Difficult | Reference | | | | | | |
|  | Easy | 0.71(0.22) | 0..28 | 1.14 | .001 | 5.93 | 2.47 | 10.06 |
|  | General | -0.06(0.23) | -0.51 | 0.39 | .79 | -0.5 | -4.31 | 3.50 |
| Symptom-specific results | 60 % | Reference | | | | | | |
|  | 70 % | -0.31(0.24) | -0.79 | 0.15 | .19 | -2.94 | -7.86 | 1.77 |
|  | 80 % | 0.69(0.27) | 0.16 | 1.21 | .01 | 5.75 | 1.48 | 10.36 |
|  | 90 % | 1.50(0.27) | 0.97 | 2.03 | <0.001 | 12.53 | 8.20 | 18.40 |
|  | 100% | 2.48(0.36) | 1.77 | 3.18 | <0.001 | 20.74 | 15.55 | 28.66 |
| Access platforms | Websites | Reference | | | | | | |
|  | App | 0.24(0.20) | -0.15 | 0.63 | .24 | 2 | -2.52 | 6.44 |
|  | WeChat applet | 0.43(0.26) | -0.09 | 0.95 | .10 | 3.62 | -0.79 | 8.10 |
|  | Hospital official accounts | 0.07(0.26) | -0.44 | 0.57 | .79 | 0.56 | -4.18 | 5.00 |
| Content model | Text | Reference | | | | | | |
|  | Phonetics | 0.48(0.26) | -0.02 | 0.99 | .06 | 4.05 | -0.35 | 8.59 |
|  | Video | 0.85(0.22) | 0.41 | 1.28 | <0.001 | 1.09 | 2.89 | 11.91 |
| Costs |  | -0.12(0.02) | 3.33 | 5.50 | <0.001 |  |  |  |

Table S2 (B) Participants with educational qualifications lower than those of a junior college

|  | Level | Preference mean difference (SE) | 95% CI | | *P* | WTP | 95% CI | |
| --- | --- | --- | --- | --- | --- | --- | --- | --- |
| Granularity | General | Reference | | | | | | |
|  | Refined | 0.145(0.33) | -0.51 | 0.80 | .66 | 6.21 | 3.24 | 9.33 |
| Linguistic comprehensibility | Difficult | Reference | | | | | | |
|  | Easy | 1.48(0.46) | 0.58 | 2.39 | .001 | 5.93 | 2.47 | 10.06 |
|  | General | 0.67(0.44) | -0.20 | 1.53 | .13 | -0.5 | -4.31 | 3.50 |
| Symptom-specific results | 60 % | Reference | | | | | | |
|  | 70 % | 0.31(0.42) | -0.52 | 1.14 | .47 | -2.94 | -7.86 | 1.77 |
|  | 80 % | 2.41(0.56) | 1.30 | 3.51 | <0.001 | 5.75 | 1.48 | 10.36 |
|  | 90 % | 3.74(0.78) | 2.21 | 5.28 | <0.001 | 12.53 | 8.20 | 18.40 |
|  | 100% | 5.69(1.06) | 3.61 | 7.78 | <0.001 | 20.74 | 15.55 | 28.66 |
| Access platforms | Websites | Reference | | | | | | |
|  | App | 0.09(0.37) | -0.63 | 0.81 | .80 | 2 | -2.52 | 6.44 |
|  | WeChat applet | 1.25(0.45) | 0.37 | 2.14 | .005 | 3.62 | -0.79 | 8.10 |
|  | Hospital official accounts | 0.73(0.51) | -0.27 | 1.73 | .15 | 0.56 | -4.18 | 5.00 |
| Content model | Text | Reference | | | | | | |
|  | Phonetics | 0.03(0.41) | -0.77 | 0.83 | .95 | 4.05 | -0.35 | 8.59 |
|  | Video | 1.13(0.42) | 0.30 | 1.95 | <0.007 | 1.09 | 2.89 | 11.91 |
| Costs |  | -0.20(0.04) | -0.28 | -0.12 | <0.001 |  |  |  |

| Level | <5,000 RMB | ≥5,000 RMB | higher educational level | lower educational level |
| --- | --- | --- | --- | --- |
| Refined | 5.43 | 1.84 | 6.21 | 6.21 |
| Easy | 4.82 | 5.1 | 5.93 | 5.93 |
| General | -0.52 | 0.61 | -0.5 | -0.5 |
| 70% | -2.15 | 3.02 | -2.94 | -2.94 |
| 80% | 6.61 | 11.29 | 5.75 | 5.75 |
| 90% | 13.24 | 16.97 | 12.53 | 12.53 |
| 100% | 20.65 | 45.32 | 20.74 | 20.74 |
| App | 2.62 | 3.59 | 2 | 2 |
| WeChat applet | 6.17 | 3.44 | 3.62 | 3.62 |
| Hospital official accounts | 4.25 | -0.56 | 0.56 | 0.56 |
| Phonetics | 1 | 0.83 | 4.05 | 4.05 |
| Video | 6.08 | 4.82 | 1.09 | 1.09 |
