# Supplementary material for: Young Adult Perspectives on Artificial Intelligence–Based Medication Counseling in China: Discrete Choice Experiment
Source: J Med Internet Res. 2025 Apr 9;27:e67744. doi: 10.2196/67744 (PMC12018864; doi:10.2196/67744)
Supplement: Multimedia Appendix 2 [file jmir_v27i1e67744_app2.doc]

**2023 Shandong Province Residents' Medication Literacy Survey A**

1. **Invitation to Participate in This Study:**

We would like to invite you to participate in an anonymous online survey, which will take approximately 15-20 minutes. This informed consent form provides information to help you decide whether to participate in the survey. Please read it carefully, and feel free to ask the researchers any questions. Your participation in this study is completely voluntary.

1. **Why Is This Study Being Conducted?**

Medication literacy is the foundation for safe medication use. This survey aims to explore the current state and factors influencing medication literacy among residents of Dongying City to improve the safety of medication use in daily life.

1. **Who Will Be Invited to Participate in This Study?**

18-44 years old;

Chinese nationality;

Able to complete the online questionnaire independently or with the assistance of a researcher;

Able to understand the meaning of each item in the questionnaire.

1. **Exclusion Criteria for This Study:**

Unconscious or mentally impaired individuals;

Individuals with cognitive impairment;

Individuals who are unwilling to participate in the study.

What Are the Risks of Participating in This Study? Some questions in the survey may make you feel uncomfortable or involve sensitive topics.

1. **What Are the Benefits of Participating in This Study?**

Your opinions on the survey will help us understand the medication literacy levels of local residents, assist in improving medication consultation services, and enhance medication safety.

1. **Is Participation Required, and Do I Have to Complete This Study?**

Participation in this study is entirely voluntary. You may choose not to participate or withdraw at any time without facing any negative consequences. We hope you will participate in this survey.

1. **Will My Information Be Kept Confidential?**

This study will strictly follow the national "Statistical Law" regarding data management and usage. The information you provide will be kept confidential. Study data will only be used for academic purposes. When the results are published, no personal data will be disclosed, and it will not negatively affect you in any way.

1. **If I Have Questions or Issues, Who Should I Contact?**

If you have any questions related to this study, please feel free to contact the study coordinator, Jia Zhang: Contact: zjiaa112@163.com

ID Number (to be filled out by the researcher)

_________________________________

I have read this informed consent form.

I had the opportunity to ask questions, and all of my questions were answered.

I understand that participation in this study is voluntary.

I can choose not to participate or withdraw at any time without facing discrimination or retaliation.

1. **If you agree to participate in our survey, please click "Agree" below to begin the questionnaire:**

○Agree (Please proceed to Question 2)

○Disagree (Please skip to the end of the questionnaire and submit your response)

**2. Your Gender:**

○Male

○Female

**3. Your Ethnicity:**

○Han Chinese

○Ethnic Minority

**4. Do You Have a Religious Belief?**

○No

○Yes

1. **Your Height (in cm):**
2. **Your Weight (in Jin):**
3. **Your Age:**

**9. Your Current Employment Status:**

○Employed

○Student

○Retired

○No Fixed Occupation (or Freelance)

○Unemployed

○Seeking Employment

**10. Your Highest Educational Level:**

○No Formal Education

○Elementary School

○Middle School

○Vocational School

○High School

○College

○Bachelor's Degree

○Master's Degree

○Doctoral Degree

**11. Where Have You Lived in the Last Three Months?**

○Urban Area

○Rural Area

**12. Your Household Registration Type:**

○Non-Agricultural

○Agricultural

**13. Your Marital Status:**

○Single

○Married (including first marriage, remarriage, or remarried)

○Divorced

○Widowed

**14. Select the Family Type that Best Fits You:**

○Couple Family (Only two people, husband and wife)

○Nuclear Family (Parents and unmarried children)

○Extended Family (Parents and married children)

○Joint Family (Parents and two or more married children or families that do not separate after marriage)

○Single-Parent Family (Single parent, divorced or widowed, with children)

○DINK Family (Couple without children by choice)

○Grandparent Family (Only grandparents and grandchildren, parents are absent)

○Single-Individual Family (Living alone after marriage age or after divorce)

○Other Family Forms (Including blended families, cohabiting families, same-sex families, etc.)

**15. Have You Lived Alone in the Last Three Months?**

○Yes

○No

**16. Your Household’s Per Capita Monthly Income:**

○≤1000 RMB

○1001-2000 RMB

○2001-3000 RMB

○3001-4000 RMB

○4001-5000 RMB

○5001-6000 RMB

○6001-9000 RMB

○9001-12000 RMB

○12001-15000 RMB

○≥15001 RMB

**17. Can You Easily Access Disease Prevention and Healthcare Services at Your Local Primary Healthcare Institutions?**

○Completely True

○Mostly True

○Somewhat True

○Completely False

Based on AI (Artificial Intelligence) medication consultation platforms, these systems function like a personal robotic general practitioner. By providing information about diseases or symptoms, you can easily obtain details about drug classifications, precautions, usage, and dosage, ensuring the rationality and accuracy of medication.

You are currently using an AI-based medication consultation. If you had to choose between two consultation products, Product A and Product B, which differ in AI medication consultation type, language comprehensibility, symptom-specific accuracy, cost, access platform, and content model, please select the product you prefer. Assume that all other conditions are the same except for the six listed attributes.

**18. Which Consultation Would You Prefer?**


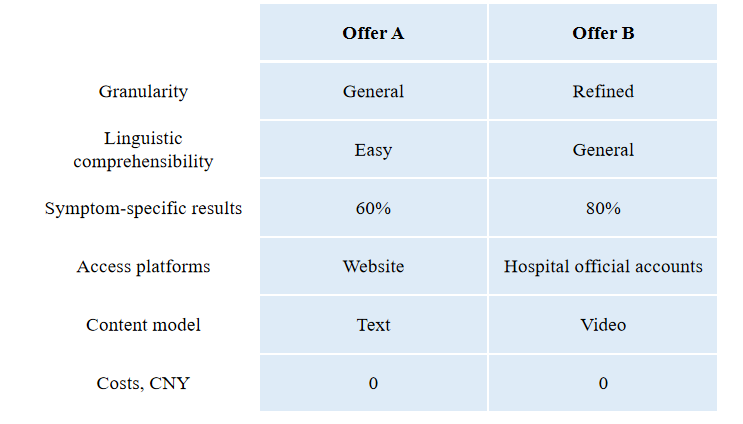


○Offer A

○Offer B

○Neither

1. **Which Consultation Would You Prefer?**


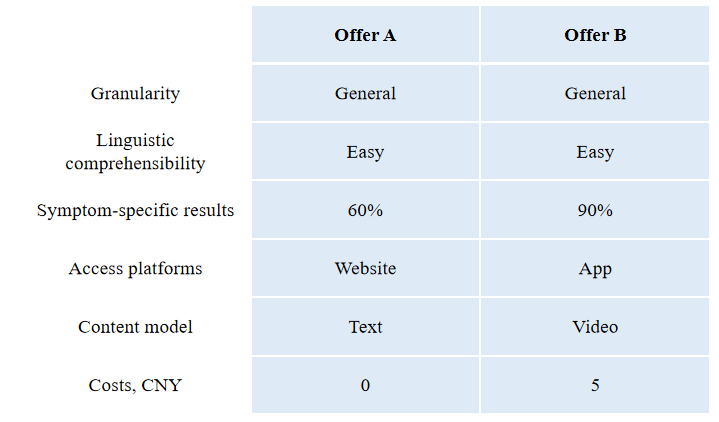


○Offer A

○Offer B

○Neither

**20. Which Consultation Would You Prefer?**


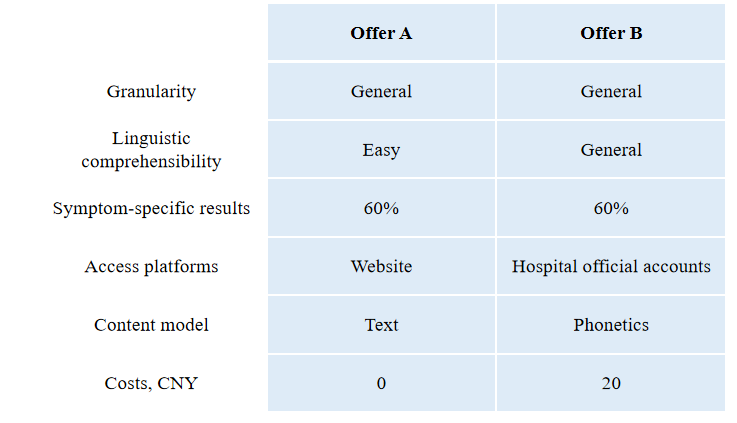


○Offer A

○Offer B

○Neither

**21. Which Consultation Would You Prefer?**


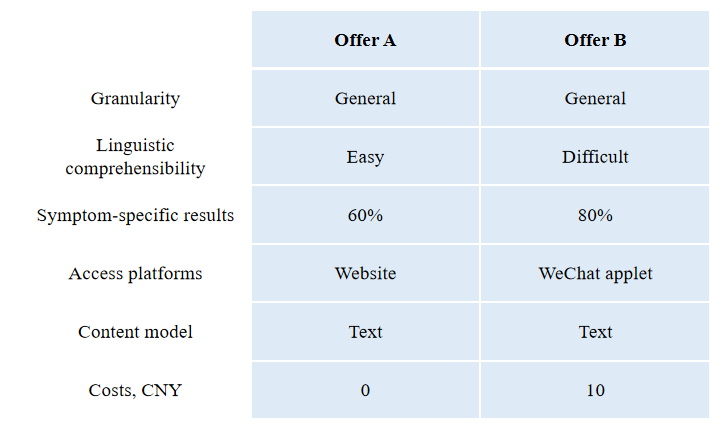


○Offer A

○Offer B

○Neither

**22. Which Consultation Would You Prefer?**


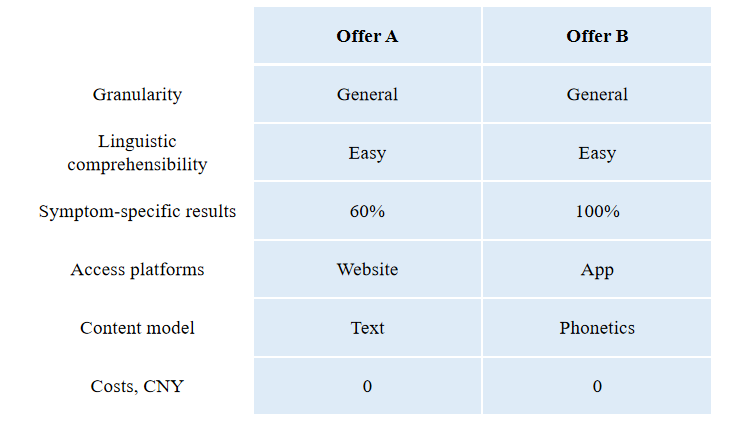
○Offer A

○Offer B

○Neither

**23. Which Consultation Would You Prefer?**


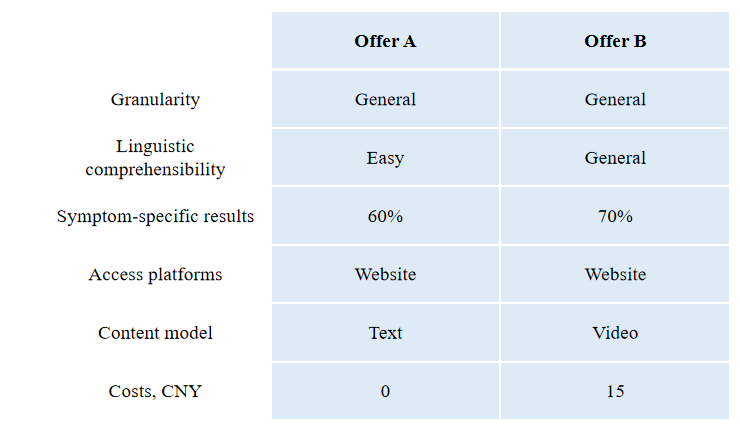
○Offer A

○Offer B

○Neither

**24. Which Consultation Would You Prefer?**


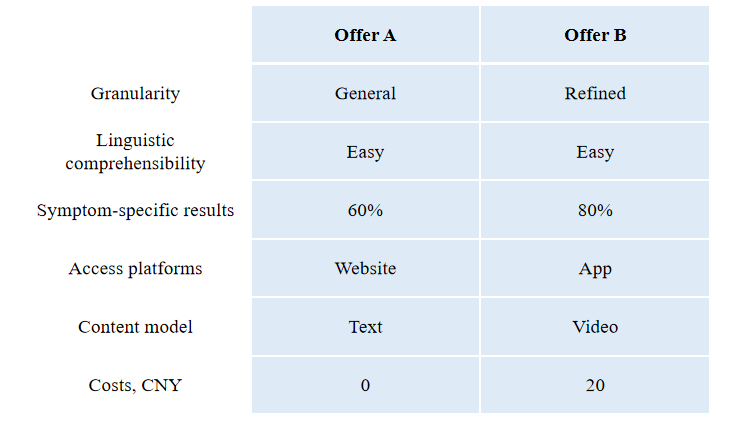


○Offer A

○Offer B

○Neither

**25. Which Consultation Would You Prefer?**


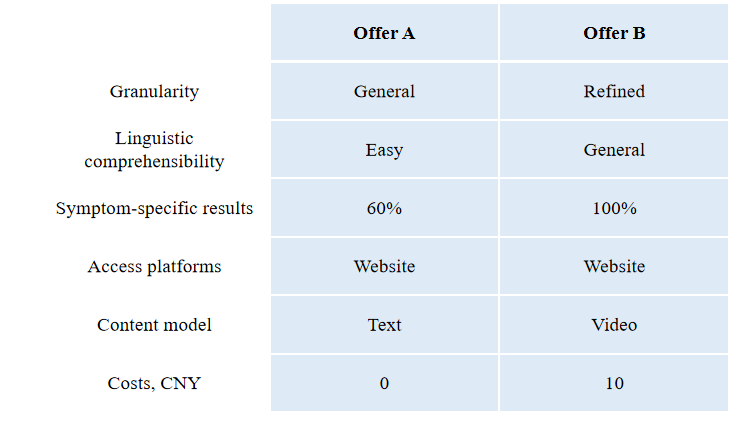
○Offer A

○Offer B

○Neither

**26. Which Consultation Would You Prefer?**


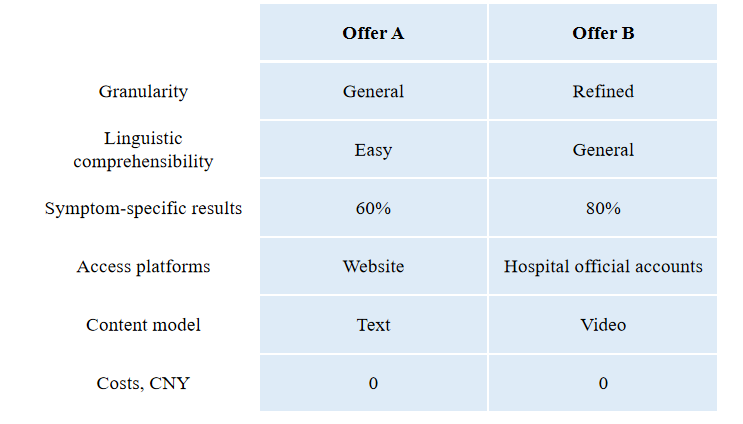
 ○Offer A

○Offer B

○Neither
